# Ökonomische Aspekte der Niederfeld-Magnetresonanztomographie: Anschaffung, Installation und Unterhaltskosten von 0,55 T-Geräten

**DOI:** 10.1007/s00117-022-00986-9

**Published:** 2022-03-29

**Authors:** Jan Vosshenrich, Hanns-Christian Breit, Michael Bach, Elmar M. Merkle

**Affiliations:** Klinik für Radiologie und Nuklearmedizin, Universitätsspital Basel, Petersgraben 4, 4031 Basel, Schweiz

**Keywords:** MRT-Gerät, Niederfeld-MRT, Kosten-Nutzen-Analyse, Energieverbrauch, Unterhaltskosten, MRI scanner, Low field MRI, Cost–benefit analysis, Energy consumption, Maintenance costs

## Abstract

**Hintergrund:**

Niederfeld-Magnetresonanztomographie(MRT)-Geräte bieten aufgrund niedrigerer Herstellungskosten und geringerer baulicher Anforderungen für Installation und Betrieb eine Chance zur Kostenreduktion.

**Fragestellung:**

Mögliche Kostenreduktionen in Anschaffung, Einbringung und Unterhalt durch die Verwendung neuer Niederfeld-MRT-Systeme.

**Material und Methoden:**

Übersicht über die wichtigsten Kostenfaktoren und Evaluierung des Einsparpotenzials eines 0,55-T-Niederfeld-MRT der neuesten Generation im Vergleich zu 1,5‑ und 3‑T-MRT-Systemen in der klinischen Routine.

**Ergebnisse:**

Im Anschaffungspreis liegt das Einsparpotenzial eines 0,55-T- im Vergleich zu einem 1,5-T-MRT-System bei etwa 40–50 %. Das um 25 % niedrigere Gewicht des Systems verringert die Transportkosten, die geringere Größe des Geräts erlaubt bei einem ebenerdigen Betriebsplatz die Einbringung mittels ferngesteuertem fahrbarem Robotersystem ohne Eröffnung der Außenfassade. Eine Quench-Leitung muss nicht installiert werden. Die Kosten für Einbringung und Installation sind insgesamt bis zu 70 % niedriger als bei Hochfeldsystemen. Die Wartungskosten eines 0,55-T-Geräts liegen etwa 45 % unter denen eines 1,5-T-Geräts bei vergleichbarem Servicevertrag. Weitere potenzielle Kostenreduktionen ergeben sich durch die geringere Raumgröße und einen potenziell geringeren Energieverbrauch für Untersuchungen und Kühlung.

**Schlussfolgerung:**

Die Verwendung von MRT-Systemen mit niedrigerer Feldstärke bieten großes ökonomisches und ökologisches Potenzial für Kliniken und Praxisbetreiber.

## Abstract (en)

**Background:**

Low-field magnetic resonance imaging (MRI) scanners offer an opportunity for cost reduction in the healthcare system. This is due to lower manufacturing costs and reduced construction requirements for installation and operation.

**Objectives:**

To discuss potential cost reductions in acquisition, installation, and maintenance by using new low-field MRI systems.

**Methods:**

We provide an overview of key cost drivers and an evaluation of the potential savings of a recent generation 0.55T low-field MRI compared to conventional 1.5T and 3T MRI systems in routine clinical practice.

**Results:**

In terms of purchase price, the savings potential of a 0.55T MRI compared to a 1.5T MRI system is about 40–50%. The 25% lower weight of the system reduces the transportation costs incurred, and the smaller size of the unit allows for installation by a remotely controlled mobile robotic system without opening the exterior façade, if the operating site is at ground level. Together with the lack of need to install a quench pipe, this reduces the total cost of installation by up to 70%. The maintenance cost of a 0.55T MRI is approximately 45% less than that of a 1.5T unit with a comparable service contract. Further cost reductions result from the smaller room size and potentially lower energy consumption for examinations and cooling.

**Conclusion:**

The use of lower field strength MRI systems offers enormous economic and environmental potential for both hospitals and practice operators, as well as for the healthcare system as a whole.

Die Gesundheitssysteme stehen weltweit unter einem gewaltigen Kostendruck. Zudem gibt es erhebliche regionale und nationale Unterschiede, sodass eine bedrückende Disparität bei der medizinischen Versorgung besteht. Es gilt daher, die Gesundheitssysteme effektiver und kostengünstiger zu machen. Einen Beitrag dazu könnten moderne Magnetresonanztomographie(MRT)-Systeme mit niedriger Feldstärke leisten, die Vorteile hinsichtlich der Herstellungs‑, Installations- und Unterhaltskosten versprechen. Unabdingbare Voraussetzung ist dabei, dass sie eine hohe diagnostische Sicherheit bieten.

Die klinische Relevanz MRT-basierter Bildgebung ist in den letzten Jahrzehnten enorm gestiegen. Der durch diese Technologie erreichte Informationsgewinn wurde in zahlreichen Studien im Bereich der Neuroradiologie [9], der muskuloskeletalen [7], der kardiothorakalen [6], aber auch der abdominellen und onkologischen Diagnostik [5] belegt. Bedingt durch die weiterhin hohen Kosten für Anschaffung, Installation und Betrieb ist die globale Verfügbarkeit der MRT für Patienten jedoch nach wie vor inhomogen und in einzelnen Regionen unverändert limitiert. Dies betrifft nicht nur Entwicklungs- und Schwellenländer, sondern auch Industrienationen [2].

Der wichtigste Faktor für den hohen Anschaffungspreis eines MRT-Geräts sind der supraleitende Magnet und die Kühlflüssigkeit, gefolgt von Kosten für die Gradienten- und Transmittersysteme. Zusammen machen diese etwa 75 % der Herstellungskosten aus [10]. Als Kühlflüssigkeit dient zumeist Helium, dessen Preis auf dem Weltmarkt enormen Schwankungen unterworfen ist [1]. Ein naheliegender Ansatz zur Kostenreduktion sind daher Systeme mit niedrigerer Feldstärke und entsprechend niedrigerem Bedarf an Kühlflüssigkeit. Weitere Kosten für den Endkunden entstehen durch den Erwerb zusätzlicher Sende- und Empfangsspulen oder Softwarepakete. Diese sind jedoch flexibel und richten sich nach dem Bedarf des Betreibers.

Das hohe Gewicht und die Größe eines Magnetresonanztomographen wirken sich erheblich auf die Kosten von Transport und Inbetriebnahme aus. So müssen in der Regel bauliche Anpassungen am Betriebsplatz vorgenommen werden. Diese setzen sich u. a. aus der Installation einer Quench-Leitung, den Kühlkomponenten und der Abschirmung des Betriebsraums vor elektromagnetischer Strahlung (Faraday-Käfig) zusammen.

Der dritte zu berücksichtigende Kostenfaktor sind die Unterhaltskosten für das installierte MRT-System. Diese setzen sich aus Wartungs- und Servicekosten, Energiebedarf für Betrieb und Kühlung sowie Personalkosten zusammen.

In diesem Beitrag soll analysiert werden, inwiefern der „total cost of ownership“ (TOC) reduziert werden kann, wenn anstelle eines herkömmlichen 1,5- oder 3‑T-MRT-Geräts ein neu entwickeltes supraleitendes 0,55-T-Niederfeld-Gerät (MAGNETOM Free.Max, Siemens Healthineers, Erlangen, Deutschland) in der routinemäßigen klinischen Versorgung zum Einsatz kommt. Die Ergebnisse stützen sich auf Erfahrungen aus der eigenen Klinik, die nicht ungeprüft auf die Situation in anderen Ländern mit unterschiedlichen Rahmenbedingungen übertragen werden können.

## Anschaffungskosten

Die Anschaffungskosten eines MRT-Geräts sind landesspezifisch und unterliegen durch mögliche Rabatte, die vom Gerätehersteller z. B. im Rahmen von Kooperationsverträgen gewährt werden können, erheblichen Schwankungen. Zudem repräsentiert der vom Betreiber zu entrichtende Endpreis meist nicht den reinen Gerätepreis, sondern spiegelt ein individuell kalkuliertes und auf die Bedürfnisse des Kunden angepasstes Paket für den Magnetresonanztomographen und weitere Komponenten wieder. Die gewählte Ausstattung des erworbenen MRT-Systems kann einerseits zusätzliche physische Komponenten enthalten, wie z. B. zusätzliche Sende- und Empfangsspulen für die Untersuchung bestimmter Körperregionen, andererseits aber auch spezielle Softwarelösungen, Sequenzen oder neu entwickelte Methoden beinhalten, z. B. im Bereich der parallelen oder funktionellen Bildgebung. Auch Softwarepakete für spezielle Nachverarbeitungstechniken der akquirierten Bilder müssen in einzelnen Fällen separat erworben werden und sind nicht in der Basisausstattung des Systems enthalten.

Reduziert auf die reinen Hardware-Komponenten eines 0,55-T-MRT-Geräts, liegen die Anschaffungskosten ca. 43 % unter denen eines im Hinblick auf Software- und Spulenausstattung vergleichbaren 1,5-T-Magnetresonanztomographen. Eine zusätzliche Kostenreduktion ergibt sich beim Transport des Geräts vom Hersteller zum Endkunden, da das Gewicht eines 0,55-T-Niederfeld-MRT-Geräts etwa 25 % geringer ist als das eines 1‑ oder 3‑T-Geräts. Für den Logistikdienstleister entspricht dies einer absoluten Gewichtsreduktion von ca. 1,1 t.

In unserer Klinik lagen die Anschaffungskosten des 0,55-T-Niederfeld-MRT-Geräts insgesamt etwa 40–50 % unter denen eines im Hinblick auf Hardware, zusätzliche Komponenten und Software vergleichbaren 1,5-T-Geräts desselben Herstellers.

## Bauliche Maßnahmen

Die Kosten für bauliche Maßnahmen und den Transport des Geräts vom Anlieferungsort durch den Logistikdienstleister bis zum endgültigen Betriebsstandort innerhalb der Klinik bzw. der Praxis unterliegen ähnlich wie die Anschaffungskosten landesspezifischen Unterschieden und sind daher ebenfalls im geografischen Kontext zu betrachten. Im Fall des Instituts der Autoren dient daher im Folgenden das MAGNETOM Prisma 3‑T-MRT-Gerät (MAGNETOM Prisma, Siemens Healthineers, Erlangen, Deutschland), welches im Jahr 2013 in einen unmittelbar angrenzenden Betriebsraum innerhalb des Universitätsklinikums eingebracht wurde, als Referenz zur Evaluation der Kosten für den Transport und die Schaffung der baulichen Betriebsvoraussetzungen.

**Figure 1:**
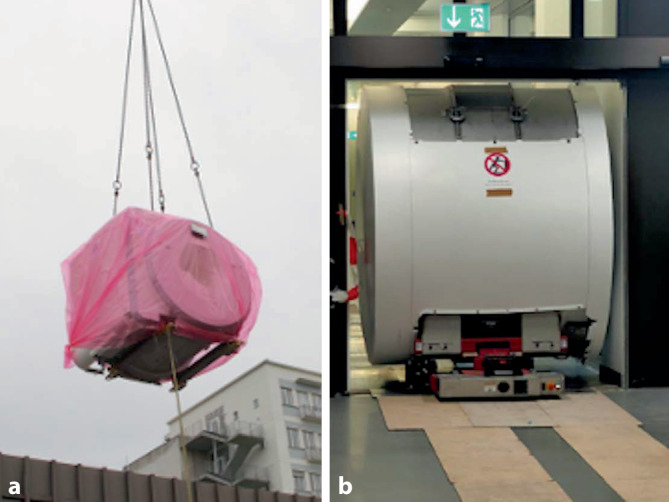

Durch das, wie bereits dargelegt, deutlich niedrigere Gesamtgewicht des 0,55-T-Niederfeld-Geräts von etwa 3,15 t gegenüber 12,25 t eines 3‑T-Scanners konnte bei der Einbringung in das Gebäude auf einen Kran verzichtet werden. Durch die geringere Transportgröße von insgesamt 230 × 165 × 198 cm (zum Vergleich 3 T: 240 × 358 × 242 cm) des neuen Magnetresonanztomographen entfiel die für die anderen im Institut installierten MRT-Scanner jeweils notwendige Eröffnung der Außenfassade des Gebäudes. Das Niederfeld-Gerät konnte per Tieflader angeliefert und mit Hilfe eines ferngesteuerten fahrbaren Robotersystems vom Eingang durch den Flur zu seinem endgültigen Betriebsplatz manövriert werden (Abb. 1). Voraussetzung für die Nutzung dieser Technik ist allerdings ein gleichgeschossiger Gebäudeeingang und Betriebsraum. Die Installation einer Quench-Leitung war aufgrund der nur sehr geringen zur Kühlung benötigten Menge Heliums von 0,7 l (im Vergleich zu je nach Typ mehr als 1000 l für herkömmlich MRT-Geräte) ebenfalls nicht notwendig.

**Figure 2:**
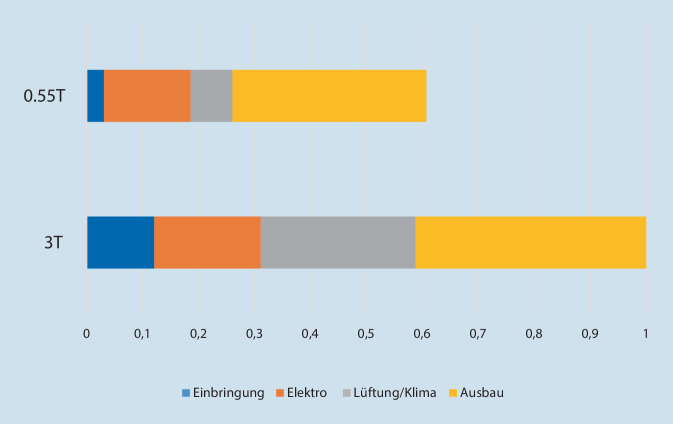

Im Vergleich zu dem vor Kurzem in unserem Institut installierten 3‑T-MAGNETOM Prisma waren die für notwendige bauliche Maßnahmen entstandenen Kosten vor der Inbetriebnahme des Niederfeld-Magnetresonanztomographen in der Summe mehr als 70 % niedriger. Dies entspricht einem mittleren sechsstelligen Betrag. Insbesondere durch die technologisch bedingt geringeren Anforderungen an Lüftungstechnik und Kühlung waren die notwendigen Kosten in diesem Bereich für das 0,55-T-Gerät etwa 73 % niedriger als für das im angrenzenden Untersuchungsraum installierten 3‑T-Gerät. Die weniger komplexen und dadurch auch weniger kostenintensiven baulichen Anforderungen in Bezug auf die elektromagnetische Abschirmung des Betriebsraums sowie die benötigten Elektroinstallationen resultierten bei den Umbaumaßnahmen für das 0,55-T-System ebenfalls in einer Kostenreduktion. Diese lagen für die baulichen Maßnahmen 16 % und bei den Elektroinstallationen 19 % unterhalb der Vergleichskosten für das 3‑T-MRT-Gerät. Eine Übersicht über die reduzierten Investitionskosten für bauliche Maßnahmen im Vergleich zu einem 3‑T-Magnetresonanztomographen ist in Abb. 2 dargestellt. In der Summe ergab sich ohne Inflationsbereinigung ein Kostenvorteil von knapp 40 % für die beschriebenen Investitionen in bauliche Maßnahmen.

## Unterhaltskosten

Die wichtigsten Faktoren bei der Betrachtung der Unterhaltskosten jedes Magnetresonanztomographen sind der Energieverbrauch (Strom und Kühlung), die Kosten für Wartungs- und Serviceverträge sowie die monatlichen Aufwendungen für den Betriebsraum. Die Wartungskosten für das 0,55-T-MRT-Gerät MAGNETOM Free.Max im Institut der Autoren liegen derzeit bei rund 55 % der Kosten für ein vergleichbares 1,5-T-System. Die Berechnung basiert auf einem in der Gesamtleistung gleichwertigen Servicevertrag.

**Figure 3:**
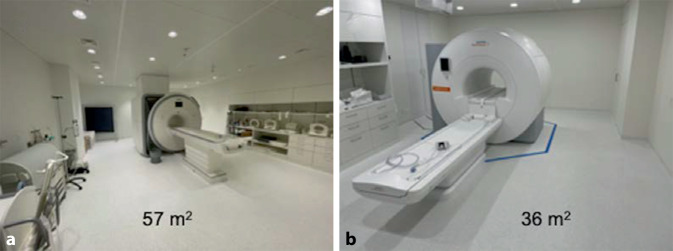

In Bezug auf die für den Betrieb benötigte Raumgröße zeigen sich erneut die Vorteile der niedrigeren Feldstärke und der verwendeten Kühlungstechnologie. Diese liegt für ein 0,55-T-Niederfeld-Gerät knapp 40 % unterhalb der für ein 3‑T-System benötigten Fläche (Abb. 3). Auch für den an den Betriebsplatz angrenzende Technikraum konnte eine Reduktion der Raumfläche von ca. 36 % erzielt werden. Vor dem Hintergrund stetig steigender Baukosten, Immobilien- und Mietpreise ist die Flächenreduktion linear mit einer Kostenreduktion um den entsprechenden Quadratmeterpreis verbunden.

**Figure 4:**
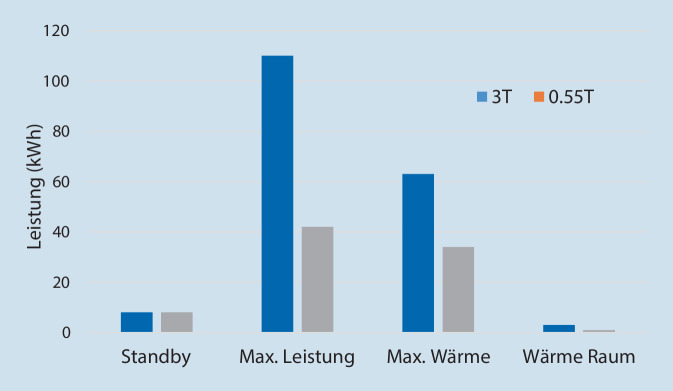

Während sich der Stromverbrauch eines Niederfeld-Scanners im Stand-by-Modus nicht von dem eines 1‑ oder 3‑T-Geräts unterscheidet, zeigt sich während der Bildakquisition ein erhebliches Einsparpotenzial. So liegt der maximale Stromverbrauch des 0,55-T-Geräts bei Betrieb unter Volllast ca. 57 % unter dem eines 3‑T-Geräts. Auch die maximale Abgabe von Energie in Form von Wärme an den Kühlkreislauf und die Umgebungsluft im Untersuchungsraum reduziert sich beim Betrieb eines 0,55-T-Magnetresonanztomographen substanziell. So beträgt die maximale Wärmeabgabe an den Kühlkreislauf nur etwa 54 % und die maximale Wärmeabgabe an die Umgebungsluft im Raum nur etwa 33 % der Werte eines 3‑T-Geräts (Abb. 4). Dies spiegelt sich in den Anforderungen an die entsprechenden Kühlsysteme und den mit der Kühlleistung verbundenen Energieverbrauch wider.

## Zukunft

Niederfeld-MRT-Geräte bieten in Bezug auf die Installationskosten und im Unterhalt gegenüber Magnetresonanztomographen mit höheren Feldstärken deutliche Vorteile, welche wir bei dem in unserem Institut installierten System in vollem Umfang bestätigt fanden.

Geringere Investitions- und Betriebskosten können die Installation von MRT-Systemen in geografischen Regionen ermöglichen, in denen Patienten bislang nur eingeschränkten Zugang zu dieser Untersuchungsmodalität und ihren Vorteilen in Diagnostik und Therapie hatten. Niedrigere bauliche Anforderungen für Installation und Betrieb eines Niederfeld-MRT-Geräts im Vergleich zu 1,5‑ oder 3‑T-Systemen, und insbesondere die für ein 0,55-T-Magnetresonanztomograhpen nicht mehr notwendige Quench-Leitung, erleichtern die Wahl möglicher Betriebsstandorte erheblich. Darüber hinaus könnte ein MRT-Gerät auch auf einer Intensivstation oder in einem Notfallzentrum installiert werden.

Ein besonderer Aspekt der betriebswirtschaftlichen Vorteile sind die damit verbundenen ökologischen Chancen. Dies insbesondere in Hinblick auf den bis dato enormen Ressourcenverbrauch, den die moderne radiologische Diagnostik mit sich bringt. Insbesondere die Komponenten des Kühlungssystems herkömmlicher MRT-Geräte tragen technologisch bedingt wesentlich zur Grundlast und zum Energieverbrauch bei, und dies im Speziellen auch außerhalb der Untersuchungszeiten im Stand-by-Betrieb [3]. Gleichwohl gilt es, noch in der Praxis zu evaluieren, ob und in welchem Maße die aufgrund der niedrigeren Feldstärke von 0,55-T-Geräten teilweise längeren Messzeiten diese Kostenvorteile konterkarieren. In der Summe sind diese Betrachtungen jedoch erneut sehr differenziert anzustellen, da sich auch die Zeit, die der Patient im Untersuchungsraum verbringt, aus verschiedenen Komponenten zusammensetzt. Hierzu zählen insbesondere aufgewendete Zeiten für Lagerung, Tischpositionierung und Kalibrierung der Messungen, die gemeinsam einen erheblichen Teil der „patient in-room time“ ausmachen [8].

Letztlich bieten Niederfeld-MRT-Geräte auch auf technischer Ebene Vorteile, die in Zukunft den Zugang zu Magnetresonanztomographien positiv beeinflussen könnten. Speziell die geringer ausgeprägten Suszeptibilitätsartefakte könnten die Untersuchung von Patienten mit metallischen Implantaten vereinfachen und die diagnostische Genauigkeit radiologischer Befunde, beispielsweise bei postoperativen Komplikationen nach der Implantation von Prothesen, erhöhen. Auch die technisch einfachere Realisierbarkeit größerer Bohrungen der Magnetresonanztomographen (z. B. 80 cm Durchmesser beim MAGNETOM Free.Max) könnte sowohl den Patientenkomfort erhöhen wie auch den Zugang zu MRT-Untersuchungen für Patientinnen und Patienten mit Platzangst erleichtern [4].

## Fazit für die Praxis

- Die Gesamtkosten eines Magnetresonanztomographen setzen sich aus Anschaffungspreis, Installations- und Unterhaltskosten zusammen.
- Der niedrigeren Anschaffungskosten für Niederfeld-MRT-Geräte basieren primär auf den niedrigeren Kosten des supraleitenden Magneten als teuerster Komponente.
- Einbringung und Installation eines Niederfeld-MRT-Scanners sind durch das geringere Gewicht und die kompaktere Bauweise ebenfalls deutlich kostengünstiger.
- Die Unterhaltskosten eines Niederfeld-MRT-Geräts zeigen ein Einsparpotenzial im Vergleich mit herkömmlichen 1,5- oder 3‑T-MRT-Systemen.
- Für eine Gesamtbilanz werden weitere Erfahrungen benötigt, ob bei feldstärkebedingt mutmaßlich längeren Messzeiten der Preisvorteil in Anschaffung, Installation und Unterhalt einen geringeren Patientendurchsatz kompensiert.
